# 36. INVESTING IN RECOVERY – AN ECONOMIC AS WELL AS MORAL IMPERATIVE

**DOI:** 10.1093/schbul/sby014.151

**Published:** 2018-04-01

**Authors:** David McDaid

**Affiliations:** London School of Economics and Political Science

## Abstract

**Overall Abstract:** ‘Recovery’ is a key concept in mental health policy around the globe. The World Health Organization has called for ‘a recovery-based approach that puts the emphasis on supporting individuals with mental disorders and psychosocial disabilities to achieve their own aspirations and goals’. Investing in evidence-based actions to help foster recovery should therefore be core to any system of support for anyone experiencing schizophrenia or other severe mental health problems. While there is clearly a moral imperative to maximise opportunities for recovery, the economic case for action can also be compelling and complementary. However, the opportunity to make an economic argument to support investment in recovery is not always taken, and even when made it is often too narrow in ambition and scope to have a major influence policy and practice. This presentation will highlight examples of the economic potential of recovery-focused services in health, employment, education and housing services. It will look at strengths and weaknesses in the way in which economic evidence is presented to policy makers, including the extent to which implementation challenges have been considered. It will argue that in making the economic case for recovery it is just as vital to look at the role of the messenger as well as the message that is being communicated.

